# Tuberous Sclerosis Complex in a 17-month-old: A Case Report

**DOI:** 10.31729/jnma.8172

**Published:** 2023-06-30

**Authors:** Sarjan K.C., Anjana Bohaju, Sunil Raja Manandhar, Anup Shrestha, Erika Aryal, Pradeep Maharjan

**Affiliations:** 1 Kathmandu Medical College and Teaching Hospital, Sinamangal, Kathmandu, Nepal; 2 Department of Paediatrics, Kathmandu Medical College and Teaching Hospital, Sinamangal, Kathmandu, Nepal

**Keywords:** *angiofibroma*, *case reports*, *seizures*, *tuberous sclerosis*, *tumor suppressor gene*

## Abstract

Tuberous sclerosis complex is a rare autosomal dominant genetic disorder that affects multiple organ systems, primarily affecting the central nervous system. It develops with a pathogenic mutation in tumour suppressor genes i.e. Tuberous Sclerosis Complex 1 or Tuberous Sclerosis Complex 2 which codes for protein hamartin and tuberin leading to unopposed hyperactivation of the mammalian target of the rapamycin signalling pathway. It presents with a triad of facial angiofibroma, intellectual disability, and epilepsy. We present a case of a 17-month female toddler with abnormal body movement with loss of consciousness and later developing into generalised jerky movements. On magnetic resonance imaging, a diagnosis of tuberous sclerosis was made. The patient underwent symptomatic management with anti-epileptic. As seizures in these cases are subtle, they remain undiagnosed for a long time leading to delays in management and developing refractory seizures.

## INTRODUCTION

Tuberous sclerosis complex (TSC) is a multi-organ involving rare autosomal dominant genetic disorder primarily affecting the brain, skin, kidney, and heart with an overall prevalence of approximately 1 in 30,000.^1^ Approximately 80% of TSC patients develop epilepsy in the first two years of life, the most common being focal seizures and infantile spasms which might get triggered by systemic illness and fever into status epilepticus.^2^ Initially, seizures in TSC are subtle which might be missed that results into refractory epilepsy.^3^ We report a case of a 17-month-old child who presented with multiple episodes of seizures and pathological findings suggestive of TSC.

## CASE REPORT

A 17-month-old female child was presented to the tertiary care centre with complaints of abnormal body movements 12 hours back which first involved stiffening of the left upper hand along with up-rolling of eyes, frothing of mouth with perioral bluish discolouration. The informant (her mother) also gave a history of loss of consciousness and unresponsiveness to vocal or tactile stimuli with clenching of teeth and urinary and stool incontinence. After a few min, abnormal body movements generalised to the right upper limb and bilateral lower limbs in the form of jerky movements that lasted for more than 30 min. She was managed with phenytoin and levetiracetam injections and then referred to our hospital for paediatric intensive care unit (PICU) admission. She also had a past history of abnormal stiffening of the bilateral upper limb with focal motor seizures of fingers since 12 months of age. She had a family history of seizure disorder with her cousin and uncle.

**Figure 1:**
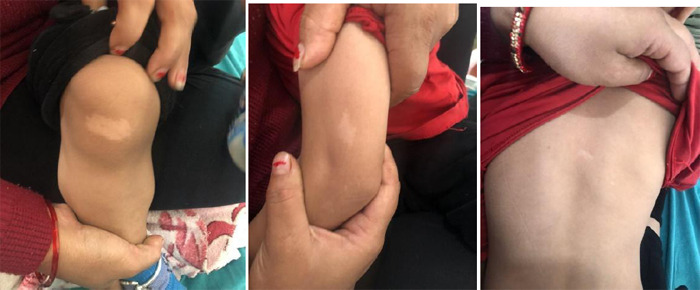
Hypopigmented macules were present over the right knee, left arm, and back.

On examination, the general condition was sedated and febrile. The pulse rate was 132 beats per min with irregular character, respiratory rate 32 times per min, body temperature 101.2°F, oxygen saturation 98% measured in the fetal monitor, and capillary refill time of fewer than 3 seconds. Hypopigmented macules were present over the forehead, right side of the face, right knee, left arm, and back (Figure 1).

**Figure 2:**
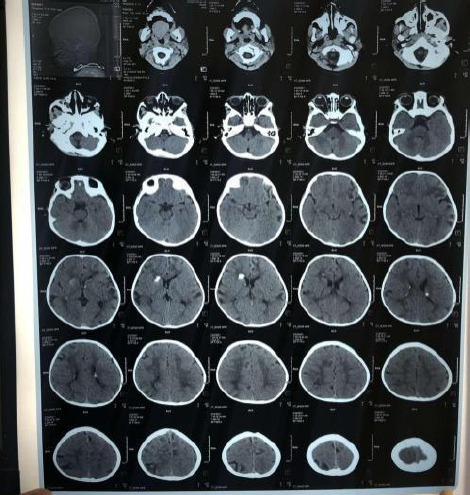
Subcortical hypodensities in the bilateral frontal temporal and parietal lobes with calcified nodules along the margin of bilateral lateral ventricles.

Routine laboratory tests were unremarkable. Computed tomography (CT) scan of the head showed subcortical hypodensities in the bilateral frontal, temporal and parietal lobes with calcified nodules along the margin of bilateral lateral ventricles (Figure 2).

Magnetic resonance imaging (MRI) of the brain revealed cortical tubers with cystic changes in the grey matter of bilateral cerebral hemispheres along with sub-ependymal nodules adjacent to bilateral lateral ventricles. Few of those nodules are calcified. Ultrasound of the abdomen and pelvis reported normal findings. Electroencephalogram (EEG) showed an interictal record of 7-10 Hz alpha activity with generalised slowing to theta and delta range. Intermittent spike slow wave complexes are seen mainly arising from the right side.

Supportive management of uncontrolled seizure was done with two anti-epileptic drugs (injection levetiracetam and injection phenytoin). CT head was done suggestive of tuberous sclerosis. The ophthalmological evaluation showed no papilledema or any retinal haemorrhages. Oral antibiotic syrup cefpodoxime was added for persistent fever and raised c-reactive protein (CRP). The patient was shifted to the ward on the third day and again shifted to PICU for 2-3 episodes of seizure. Injection sodium valproate was added. After 2 days of seizure-free episodes, the patient was shifted to the ward and discharged on the eighth day of admission.

At the time of discharge, the patient was seizure free and afebrile for more than 3 days and was tolerating oral feed without abdominal distension and vomiting and is passing stool and urine normally She was recovering well with a normal state of health as stated by investigation reports. She was prescribed syrup cefpodoxime 2.5 ml twice a day for 2 days, syrup vitamin B-complex 5 ml once a day for 2 weeks, and advised to continue syrup sodium valproate 3 ml, tablet phenytoin 40 mg and syrup levetiracetam 3 ml per oral twice a day. And to use aerosol midazolam 2 puffs intranasal during seizure attacks. The patient was then discharged with advice for follow-up in the pediatric outpatient department (OPD) after 4 days with EEG and MRI brain report and tapering of phenytoin on follow-up. Counselling was done with a proper explanation of danger signs to the informant. On follow-up, an MRI brain report showed cystic changes in bilateral cerebral hemispheres for which she was advised to drug continuation. The patient party was advised for follow-up in the duration of 3 months regularly. According to the follow-up, she is within a normal state of health and is in continuation of the drugs prescribed to date.

## DISCUSSION

TSC is also known as epiloia or pringle-bourneville phacomatosis.^4^ Mostly TSC shows a triad of facial angiofibroma, intellectual disability, and epilepsy.^5^ It develops with a mutation in tumor suppressor genes i.e. TSC1 (chromosome 9q34)^6^ and TSC2 genes (chromosome 16p13.3)^6^ which codes for hamartin and tuberin respectively that leads to permanent activation of the mammalian target of the rapamycin (mTOR) pathway affecting cellular proliferation^4^ which later results into dysfunction of affected organs with hamartomas and focal dysplastic lesions formation.^7^ TSC2 genes are more common to be involved with more severe neurological dysfunction as compared to TSC1 gene which can be identified by genetic testing.^4,7^ Almost two-thirds of all cases of TSC are sporadic type but in our case, it is familial in origin.^1^

Most patients present with TSC as a newborn or as a young child as seen in this case but sometimes they develop elusive symptoms which are not diagnosed till adulthood.^8^ As according to the International tuberous sclerosis complex consensus conference 2012 diagnostic criteria for TSC comprises two major features or one major feature with two or more minor features.^9^ Similar to that in our case the patient presented with three major features i.e. more than three hypomelanotic macules which were greater than 5 mm in diameter, cortical tubers with cystic changes in bilateral cerebral hemispheres along with bilateral sub ependymal nodules adjacent to bilateral lateral ventricles and was diagnosed of tuberous sclerosis.

The most prevalent presentation of TSC is epilepsy ^10^, which might progress into status epilepticus (SE) or refractory seizure.^11^ Different risk factors are associated with progressions like non-compliance to treatment, fatigue, infections, intellectual disability, and symptomatic epilepsy.^11^ SE and refractory seizure are usually associated with increased neurological and behavioural morbidity and mortality requiring urgent medical care.^10^ However, there is very little literature on TSC-associated SE, and among those very few were of non-convulsive SE (NCSE). Two cases of TSC-associated NSCE were reported in 2019.^11^ In our case the patient presented with NCSE initially which later on progressed to generalized convulsive seizure.

Antiepileptic treatments are recommended after two unprovoked clinical seizures or after one seizure in patients at high risk of recurrent seizures.^12^ Immediate treatment with anti-epileptic after seizure-onset in children decreases the risk of neurodevelopmental complications but about 50-60% of them may develop intellectual disability further.^12^ A case of epilepsy-associated TSC was reported in 2022 where levetiracetam was given for the management of epilepsy.^13^ Similar to that, in our case patient was treated with levetiracetam along with phenytoin as soon as possible to prevent developing complications. Counselling of the patient party was done about the risk of seizures and suggested to visit a neurologist when seizures occurred. Each lesion or symptom is highly variable with respect to age,^6,7^ onset, and severity of organ involvement so the management of TSC patients requires life-long follow-up.^7^

With the absence of the facility of genetic testing, we had limitations that could not distinguish the mutated gene (i.e. TSC 1 or 2) in our patient. As there might be subtle symptoms presented in patients so it can be underdiagnosed till adulthood when the seizures could develop into a refractory type. So during investigations, the seizure presented should be assumed to be repeated, and the history of previous seizure episodes should be asked thoroughly for appropriate treatment.
